# Trained immunity in type 2 immune responses

**DOI:** 10.1038/s41385-022-00557-0

**Published:** 2022-09-05

**Authors:** Franziska Hartung, Julia Esser-von Bieren

**Affiliations:** Center of Allergy and Environment (ZAUM), Technical University of Munich and Helmholtz Center Munich, 80802 Munich, Germany

## Abstract

Immunological memory of innate immune cells, also termed “trained immunity”, allows for cross-protection against distinct pathogens, but may also drive chronic inflammation. Recent studies have shown that memory responses associated with type 2 immunity do not solely rely on adaptive immune cells, such as T- and B cells, but also involve the innate immune system and epithelial cells. Memory responses have been described for monocytes, macrophages and airway epithelial cells of asthmatic patients as well as for macrophages and group 2 innate lymphoid cells (ILC2) from allergen-sensitized or helminth-infected mice. The metabolic and epigenetic mechanisms that mediate allergen- or helminth-induced reprogramming of innate immune cells are only beginning to be uncovered. Trained immunity has been implicated in helminth-driven immune regulation and allergen-specific immunotherapy, suggesting its exploitation in future therapies. Here, we discuss recent advances and key remaining questions regarding the mechanisms and functions of trained type 2 immunity in infection and inflammation.

## Introduction

The concept of “trained immunity” in humans was first proposed in the context of vaccination against tuberculosis with Bacillus Calmette-Guérin (BCG)^1^, which provides protection against other infections^2,3^ as well as against unrelated pathologies, including bladder cancer^4,5^. Trained immunity is not unique to mammals, but represents the only possibility to generate immunological memory in the absence of an adaptive immune system, e.g. in plants and invertebrates^1^. Trained immunity, also called “innate memory” describes the concept that - upon encounter of an infectious or inflammatory agent - innate immune cells undergo epigenetic reprogramming, which alters the cells’ responsiveness to subsequent unrelated insults. The mechanisms and functions of trained immunity are particularly well-characterized for monocytes and macrophages, which acquire enhanced effector functions against bacterial, viral or fungal infections following BCG vaccination or exposure to pathogen molecules such as beta-glucan^2,6–9^. Trained immunity induced by viral infections has also been described for natural killer (NK) cells^10,11^, which display receptor-dependent pathogen specific memory effects that resemble T cell memory responses^12^.

Major mechanisms underlying trained immunity include metabolic and epigenetic rewiring, which is particularly well-described for monocytes^6–8^ and their progenitors in the bone marrow (BM)^9,13^. Epigenetic reprogramming of hematopoietic stem and progenitor cells (HSPCs) in the BM, called “central trained immunity” provides particularly long-lasting effects on immunity and inflammation. Similarly, progenitor cells at epithelial barriers (e.g. basal progenitors of airway epithelial cells) can undergo epigenetic and transcriptional reprogramming, thus potentially perpetuating chronic inflammation locally^14^. The concept of long-term inflammatory reprogramming affecting both innate immune cells, structural cells (e.g. epithelial cells and fibroblasts) and their progenitors is referred to as “inflammatory memory”^15^.

On the other hand, the anti-inflammatory reprogramming of epithelial cells may confer long-term protection against allergic airway inflammation (AAI) (e.g. in farming environments) by shutting down TNF-driven inflammatory memory responses^16^ via immune regulatory factors such as A20^17^.

In addition to its directs effects on host immunity, trained immunity (e.g. to fungal infection) may even be transmitted to the offspring—at least under certain conditions—which still need to be fully resolved^18,19^. Several lines of evidence in mouse models of infection or inflammation as well as in humans suggest that maternal infection or inflammatory disease can result in an altered immune state in offspring, associated with enhanced cytokine responses of innate immune cells and epigenetic reprogramming of epithelial stem cells^20–22^. Discrepant findings on the vertical transmission of trained immunity may be explained by environmental factors such as the maternal microbiome, which may also influence a potential transmission of allergen- or helminth-induced trained immunity to the offspring^23,24^. While the mechanisms and functions of trained immunity in the context of bacterial and fungal infections are relatively well understood, the mechanistic underpinnings and consequences of trained immunity are only beginning to emerge for other diseases, including cancer, cardiovascular disease or chronic inflammatory conditions. This is particularly true for type 2 immune responses, which play important roles in anti-helminth immunity and diseases such as allergy and asthma.

While type 2 immune responses enable host defense against helminth parasites as well as wound healing, their aberrant and chronic activation results in inflammatory diseases such as allergy, asthma or nasal polyposis. Epidemiological evidence suggests long-lasting and non-specific effects of type 2 immunity on heterologous immune responses in humans, which may e.g. result in compromised anti-bacterial immunity in asthmatic patients^25–27^. In addition, helminth infection can negatively regulate host defense against *Mycobacterium tuberculosis*, while being protective against the development of diabetes^28,29^. Data from in vitro studies with human cells and animal models support key roles of innate immune cells in the effects of helminth infection or asthma on the subsequent susceptibility to autoimmunity or infections with respiratory viruses or helminths^30–35^.

Epithelial cells, particularly tuft cells, ILC2, neutrophils, eosinophils, mast cells (MCs) and macrophages represent key players in the innate immune response to helminth infection and allergenic stimuli. Upon encountering allergens or helminth products, epithelial cells release alarmins (IL-25, IL-33, thymic stromal lymphopoietin (TSLP)) and cysteinyl leukotrienes (cysLTs), which stimulate the recruitment, activation and expansion of ILC2^36–39^. Type 2 cytokines (IL-4, IL-5, IL-13) produced by activated ILC2 then stimulate the recruitment, activation and expansion of eosinophils and alternatively activated macrophages (AAM), which mediate helminth killing and trapping as well as tissue repair^31,40–43^.

Only few studies have investigated the role of trained immunity in host defense against helminth parasites or in the pathogenesis of chronic type 2 inflammation. Here, we provide an overview of the cellular players and molecular mechanisms that contribute to trained immunity in type 2 immune responses. Throughout this review, we use the term “immune memory” in the context of memory T- and B cells and “trained immunity” or “innate (immune) memory” when referring to innate immune- or epithelial cells. We discuss important remaining questions related to the functions, duration and mechanisms of trained type 2 immunity and highlight key issues that should be addressed in future studies. We further highlight potential parallels to trained immunity in “non type 2” settings, which may provide clues for the emerging field of “trained type 2 immunity”.

## Trained type 2 immunity induced by helminths

### Trained immunity in host defense against helminths

While the contribution of trained immunity to anti-helminth immunity in humans remains unclear, several studies in mice suggest that macrophages and ILC2 can be reprogrammed to provide enhanced protection against helminth parasites (Table 1).

**Table 1:** Triggers, mechanisms and functions of trained immunity or inflammatory memory in type 2 immune responses.

Trigger	Mechanism	Functional effects	References
Helminth infection			
*N. brasiliensis* infection	IL-13 production by neutrophils; IL4Rα-dependent alternative macrophage activation	Enhanced macrophage-mediated killing of *Nb* larvae, enhanced expression of integrins, Arginase-1 and CCL17	^45^
*S. venezuelensis* infection	IL-33-dependent ILC2 expansion & activation; eosinophil activation	Reduced worm burdens in subsequent *Nb* infection	^31, 61^
	IL-4 and IL-13 production by eosinophils	Prevention of neuronal loss during subsequent enteric infections	
House dust mite extract	Eosinophil expansion and activation (CD4^+^ T cell dependent)	Improved host defense against *Ascaris* infection	^35^
*F. hepatica* products	Reprogramming of myeloid progenitors in the bone marrow	Long-lasting protection against EAE	^30, 48^
Type 2 inflammation (allergy, asthma, CRSwNP)			
? in non-allergic asthmatics	Reduced DNA methylation, transcriptional reprogramming and aberrant lipid/acylcarnitine metabolism in human MDM	Chronic type 2 airway inflammation by increased CXCL8, CCL20, LTB_4_ and FAO?	^53^
House dust mite extract	TNF-dependent reprogramming of human MDM and murine bone marrow progenitors	Enhanced cysLT and CCL17 secretion in type 2 airway inflammation	^16^
IL-33	Trained ILC2 display genetic profile similar to memory CD4^+^ T cells, STAT3-driven epigenetic remodeling?	Increased IL-5, IL-13 secretion, promote Th2 differentiation	^47, 113^
IL-13	Epithelial stem cells epigenetically reprogrammed by IL-13?	Disruption of epithelial barrier, chronic type 2 airway inflammation; nasal polyposis?	^14^
AIT	Tolerance induction of ILC2, monocytes, DCs	Switch to ILC1, increase in anti-inflammatory monocytes and pDCs	^104^

### Group 2 innate lymphoid cells as mediators of trained immunity during helminth infection

Non-specific, ILC2- and eosinophil-mediated host defense against the rodent hookworm parasite *Nippostrongylus brasiliensis* (*Nb*) is observed in mice that have been previously infected with *Strongyloides venezuelensis* (*Sv*), another rodent parasite^31^. Despite highly similar lifecycles of these two parasites, which first infect the skin, then the lung and finally the intestine of mice and rats, *Sv*-induced protection against *Nb* is only directed against the lung stage, i.e. the larvae residing in the lung between day 1 and day 3 post infection. Protective effects depend on ILC2 and eosinophils as *Sv*-induced increases in type 2 cytokines in the lung as well as decreases in *Nb* worm burdens are abrogated in mice that lack ILC2 or eosinophils. In contrast, the protective effects of a previous *Sv* infection remain intact following depletion of CD4^+^ T cells, suggesting that—at least during the initial weeks following helminth infection—heterologous protection against other nematodes is independent of adaptive immunity.

Importantly, previous *Sv* infection can protect mice against *Nb* infection for at least 3 months, which may indicate the induction of long-lasting trained immunity programs in ILC2 and/or eosinophils. However, the epigenetic and transcriptional profiles of nematode/type 2-trained ILC2 or eosinophils remain to be defined. Despite showing correlations between ILC2 numbers, type 2 cytokine responses and *Sv-*induced long-term protection against *Nb*, enhanced host defense at later time points may at least in part depend on cross-reactive Th2 cells, which respond to a common antigen of both nematodes. Indeed, an increased antigen-specific Th2 response may explain the persistent increase in eosinophil numbers and drive AAM activation, which is essential for host defense against *Nb*^44^.

### Trained immunity as a mechanism of macrophage-mediated host defense against helminths

**Fig. 1:**
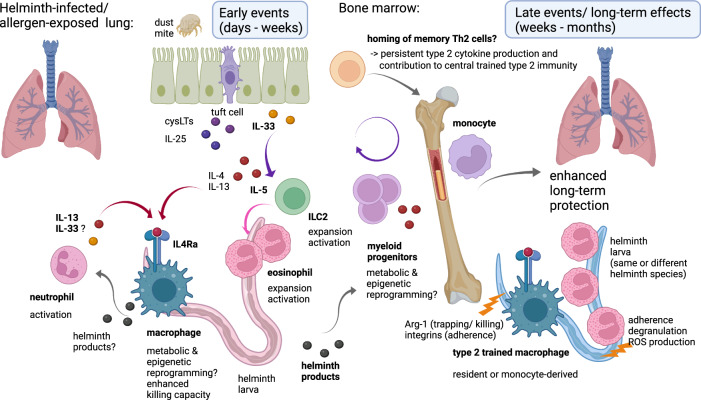
Mechanisms and functions of trained immunity in helminth infection. Created with BioRender.com.

Importantly, macrophages from the lungs of *Nb*-infected mice can transfer protection to naïve mice when isolated more than one month post infection^45^. This suggests a stable type 2 imprinting in resident and/or recruited macrophages^46^, which is maintained even following transfer into naïve lungs. In vitro, macrophages from the lungs of mice that have been infected with *Nb* for 7 or 45 days, efficiently adhere to and kill *Nb* larvae, while macrophages from naïve lungs do not show anti-helminthic effects. This suggests the rapid and long-lasting induction of a macrophage phenotype with anti-helminth effector functions, which largely depends on innate immune priming as macrophages isolated early or late after helminth infection show similar capacities to kill parasites. Indeed, neutrophil depletion abrogates the elicitation of protective macrophages by *Nb*, suggesting that the crosstalk between granulocytes and other innate immune cells, including ILC2^31^ and macrophages^45^ is centrally involved in the induction of trained immunity during helminth infection (Fig. 1). While neutrophils likely act as early inducers of trained type 2 immunity, eosinophils represent important effector cells at later time points, when type 2 activated neutrophils, ILC and macrophages have generated the type 2 inflammatory milieu that recruits, expands and activates these cells.

### Eosinophil-mediated trained immunity against helminths

A heightened eosinophil response induced by a previous, distinct type 2 immune trigger (house dust mite (HDM)) also provides protection against subsequent infection with *Ascaris*, supporting a role for trained granulocytes in non-specific, type 2-mediated host defense against helminths^35^. However, depletion of CD4^+^ T cells abrogates the heightened eosinophil response in the lungs of mice previously sensitized to HDM, suggesting that the allergen-induced eosinophil-mediated protection against *Ascaris* infection largely depends on Th2 cells. As *Ascaris* inoculation in this study is performed immediately following allergen sensitization, it is difficult to discern whether the long-term reprogramming of innate immune cells, which is observed in HDM-induced AAI^16,47^ may contribute to the protective effects of previous allergen sensitization against helminth infection.

### Type 2 inflammatory memory in different barrier tissues

As current studies investigating trained immunity in helminth infection have largely focused on the lung, it will be important to study whether previous helminth infection or allergen sensitization can induce trained immunity in distinct barrier tissues. Long-term effects of AAI or helminth products on BM progenitors^16,30,48^ indeed suggest the induction of central trained immunity programs with the potential to shape subsequent immunity in tissues with homeostatic or inflammation-induced replenishment of myeloid cell populations such as the intestine^49^, lung^46,50^ or skin^51^. Indeed, the induction, mechanisms and functions of trained type 2 immunity in barrier organs such as the skin or intestine are only beginning to emerge^15^. An inflammatory memory has recently been suggested for monocytes from food (egg) allergic children, which showed exaggerated inflammatory cytokine responses upon LPS stimulation^52^. Specifically, LPS-stimulated monocytes of food allergic infants showed an increased LPS-driven induction of TNF and IL1β, which is in line with the increased induction of these cytokines in monocyte-derived macrophages of asthmatics^16,53^. It will be highly interesting and important to delineate and compare the characteristics, mechanisms and consequences of trained type 2 immunity during helminth infection or allergic diseases at distinct barrier sites.

### Role of adaptive immune cells in trained immunity during helminth infection

Since a potential crosstalk between trained innate immune cells and the adaptive arm of anti-helminth immunity (particularly Th2 cells and B cells/antibodies) likely contributes to the maintenance of host protective trained immunity, interactions between innate and adaptive immune cells need more detailed investigation particularly at later time points after helminth infection or after challenge infection. This is particularly relevant for helminth parasites that establish chronic infection during primary infection but are expelled or killed by an antigen specific Th2 response during challenge infection. It will also be interesting to compare trained immunity responses during infection with nematodes such as *Sv* and *Nb* that are rapidly expelled to potential trained immunity programs during infection with parasites that suppress type 2 immunity and cause chronic infection (e.g. *Heligmosomoides polygyrus bakeri* (*Hpb*)).

### Induction of trained immunity by helminth products

Recent studies suggest that *Hpb* and its products affect the metabolism and effector functions of tuft cells, ILC2, eosinophils, neutrophils and macrophages, which play key roles in the early type 2 immune response against helminths^54–56^. For example, excretory secretory products of adult *Hpb* parasites inhibit the IL-4/IL-13-triggered expansion of tuft cells during helminth infection, suggesting that helminth molecules can interfere with the development of a type 2 inflammatory epithelial memory^55^. Two recently identified *Hpb* molecules (*H. polygyrus* Alarmin Release Inhibitor (HpARI) and *Hpb* glutamate dehydrogenase (*Hpb* GDH) may also affect trained immunity during allergy or helminth infection by inhibiting IL-33-driven ILC2 and eosinophil responses or type 2 driving myeloid eicosanoid responses, respectively^54,56^.

Indeed, products of the trematode *Fasciola hepatica* (*Fh*) induce anti-inflammatory trained immunity in long-term hematopoietic stem cells (HSCs) and monocyte precursors^30^, indicating that helminths can induce long-lasting immune regulatory trained immunity programs, which prevent the development of inflammatory diseases. Thus, while the type 2 immune response triggered by helminth infection or allergens induces a proinflammatory macrophage phenotype with enhanced type 2 effector functions (e.g. CCL17 or cysLT production)^16,45^, helminth molecules may counteract the inflammatory imprinting of macrophages to enable evasion from host immunity.

In particular, *Fh* products trigger myelopoiesis, resulting in the expansion of macrophage progenitors that can differentiate into immune regulatory BM-derived macrophages (BMDM) in vitro. While *Fh*-trained myeloid cells prevent the development of experimental autoimmune encephalomyelitis (EAE), a mouse model of multiple sclerosis, the exact mechanisms that mediate this protection remain to be identified. Blunted cytokine responses following in vitro treatment of *Fh*-trained BM cells with inhibitors of mTOR and HDACs suggest roles for metabolic and epigenetic reprogramming, but information about metabolic signatures or chromatin accessibility of helminth-trained myeloid cells is currently lacking.

In addition, the exact molecules responsible for the helminth-mediated induction of trained immunity remain to be identified. Helminths express several molecules that have the potential to directly or indirectly induce metabolic and epigenetic reprogramming of innate immune cells. This includes beta-glucan^57^, a well-characterized trigger of trained immunity^8^ as well as proteins targeting IL-33^54,58,59^ or metabolic enzymes, which may directly influence the metabolism of innate immune cells^56^. In addition, filarial nematodes can harbor endosymbionts, which can functionally reprogram macrophages towards an immune regulatory phenotype^60^. Thus, helminths have a broad potential to regulate inflammatory memory responses and future studies should clarify how individual helminth-derived molecules may shape immunity and inflammation over prolonged periods of time.

### Roles of type 2 cytokines in trained immunity during helminth infection

### IL-4Rα-mediated macrophage training during helminth infection

Both IL-13 and IL-33 have been implicated in type 2 imprinting of macrophages or ILC2 during helminth infection, respectively^31,45^. During infection with *Nb*, macrophages are primed for enhanced parasite killing in an IL-4 receptor alpha (IL-4Rα) dependent manner^45^, suggesting that IL-4 and/ or IL-13 drive reprogramming towards a phenotype with enhanced type 2 effector functions (Fig. 1). Indeed, early IL-13 production by neutrophils is required for this anti-helminthic macrophage training as neutrophil depletion abrogates the rapid IL-13 response observed after *Nb* infection as well as long-lasting enhanced AAM gene expression and effector functions. Specifically, lung macrophages trained by IL-13-producing neutrophils show increased expression of AAM markers (*Arg1*, *Chi3l3*, *Mgl2*, *Clec4a2* and *Ccl17*) and integrins 7 days post *Nb* infection, associated with an enhanced capacity to adhere to and kill helminth larvae for up to 45 days after inoculation^45^. Thus, gene expression profiles of macrophages imprinted during helminth infection at least partially overlap with profiles of allergen-trained macrophages, which also show increased CCL17 responses and integrin expression^16^. Eosinophils represent an additional innate source of IL-4 and IL-13 and eosinophil-derived IL-4 and IL-13 mediate *Sv*-induced long-term protection against neuronal loss triggered by unrelated enteric pathogens via activating Arginase-1-expressing AAMs^61^.

### Alarmins as “entrainers” during helminth infection

In addition to producing IL-13, neutrophils may contribute to IL-33 production^45^, thus activating IL-13 and IL-4 production by ILC2 and potentiating IL4Rα-mediated AAM priming. Indeed, IL-33 is suggested to contribute to the ILC2-dependent *Sv*-induced protection against a subsequent infection with *Nb*^31^. As IL-33 is usually considered to be released by epithelial cells, stromal cells^62–64^ and dendritic cells^65,66^, it would be important to determine the relative contribution of neutrophil-derived IL-33 to reprogramming of ILC2 and macrophages in different settings of type 2 immunity. One possibility is that neutrophils (together with mast cells) contribute to the protease-mediated processing and thus activation of IL-33^67,68^. In addition to IL-33, IL-25, which triggers the expansion and migration of inflammatory ILC2 in the small intestine during helminth infection, may represent a potential entrainer of ILC2 with enhanced type 2 cytokine production and anti-helminth effector functions^69,70^. While alarmins and type 2 cytokines likely play important roles in the induction of trained type 2 immunity, it is currently unclear whether these factors alone can induce long-term reprogramming of innate immune cells involved in host defense or tissue repair. IL-4 and IL-33 indeed have profound effects on the metabolism and epigenetic signature of macrophages^71–74^, but whether the same pathways govern trained immunity during helminth infection requires further investigation.

### Helminth-induced trained immunity in humans

Little is known about long-term changes in the innate immune cell compartments of helminth-infected humans as well as underlying mechanisms and functional consequences. Helminth-infected individuals show changes in hematopoiesis^75^ as well as aberrant monocyte^76^ and ILC2^77^ responses, which may be indicative of trained immunity. However, studies in human cohorts have shown that deworming abrogates regulatory effects of helminth infection on heterologous immune responses in infection and inflammation, suggesting that helminth-induced trained immunity wanes several months after the infection^78–80^. Thus, the intensity and duration of the infection as well as the immune response mounted against a specific pathogen will likely determine the cellular players and persistence of trained immunity in helminth-infected individuals. Given the potent effects of helminths and their molecules on immunity and inflammation, it will be important to determine the functional contribution and mechanistic basis of trained type 2 immunity in human helminth infection.

### Potential effects of helminth-induced trained immunity on inflammatory diseases

As type 2 cytokines are upregulated in the bone marrow of *Fh-*trained mice^30^—similar to observations in HDM-sensitized mice^16^—it would be important to determine the effects of helminth-induced trained immunity on distinct type 2 immune settings, e.g. in allergy. Given that IL-4 and IL-5 have been implicated in protective effects of helminth products against EAE^81,82^, a potential contribution of type 2 cytokine production by bone marrow (BM) cells to the long-lasting, helminth-induced protection against EAE should be investigated. Finally, the roles of type 2 cytokines and alarmins that are produced in local tissues or in the BM of helminth-infected mice in the induction and maintenance of trained type 2 immunity need to be clarified.

## Inflammatory memory in allergy and asthma

In contrast to its protective effects in infectious diseases, trained immunity may contribute to pathology, disease progression and chronicity in inflammatory diseases^83–85^. For example, sterile inflammatory triggers e.g. oxidized low-density lipoprotein (oxLDL) particles or western diet can induce trained immunity in the context of atherosclerosis via activation of the NLRP3 inflammasome and epigenetic reprogramming of monocytes and myeloid progenitors^83,86^. Inflammasome activation has also been implicated in the development and exacerbation of asthma^87–89^, however the inflammatory memory programs that may drive type 2 inflammatory diseases are only beginning to be uncovered.

Asthma is a heterogeneous inflammatory disorder characterized by acute bronchospasm in response to triggers such as allergens, cold air or exercise as well as chronic airway inflammation and remodeling^90^. There are several forms of asthma including allergic asthma evoked by allergens such as house dust mite^91^, pollen^92^, pollutants^93^ and molds^94^; and non-allergic asthma, which may be triggered and exacerbated by obesity^95^, cigarette smoke^96^, and nonsteroidal anti-inflammatory drugs (NSAIDs)^97^. NSAID intolerant asthma is commonly occurring together with chronic rhinosinusitis with nasal polyposis (CRSwNP), which is driven by type 2 inflammation in the nasal mucosa. Recent studies have shown that allergic as well as non-allergic, NSAID intolerant asthma as well as CRSwNP are associated with metabolic, epigenetic and transcriptional reprogramming of macrophages, ILC2 and/or epithelial cells^14,16,47,53^ (Table 1).

### Inflammatory epithelial memory in chronic type 2 airway inflammation

As key “first-responder” cells at mucosal barriers, airway epithelial cells orchestrate the induction of innate type 2 inflammation by responding to allergens and producing alarmins and cysteinyl leukotrienes (cysLTs), which stimulate ILC2 activation^37–39,98^. In turn, IL-13 derived from ILC2, acts on airway epithelial cells and disrupts epithelial barrier integrity, thus further perpetuating type 2 airway inflammation^99^. In addition to these acute effects, IL-13 exposure can alter DNA methylation and transcriptional profiles of airway epithelial cells, resulting in the upregulation of fibrotic and inflammatory pathways^100^. Differential methylation of IL-13-responsive CpG sites is apparent in freshly isolated airway epithelial cells from asthmatic and non-asthmatic individuals^100^. However, whether this altered epigenetic state is long-lasting and extends also to epithelial stem cells in asthmatic airways needs further investigation. Single cell RNA sequencing has shown that IL-13 induces an enhanced mucus secretory expression profile in all airway epithelial cell types. Chronic IL-13 exposure results in a proteome which suppresses mucociliary transport potentially due to dysfunctional cilia or to changes in mucus composition, thus reducing innate airway responses. The IL-13-mediated epithelial remodeling described in vitro is reflected by transcriptional profiles of nasal epithelial cells of asthmatic children^101^. Despite these proinflammatory effects on airway epithelial cells, whether IL-13 may induce a persistent type 2 imprint in epithelial stem cells in asthma or CRSwNP remains elusive.

**Fig. 2:**
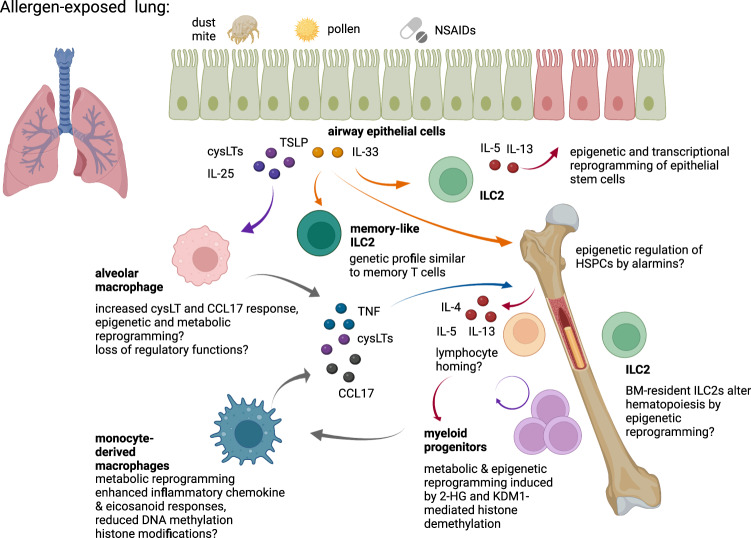
Pathological effects and mechanisms of inflammatory memory in chronic type 2 inflammation. Created with BioRender.com.

Insights into trained immunity in epithelial progenitor cells, especially, basal progenitor cells which give rise to specialized epithelial cells have been provided by single cell RNA sequencing of nasal polyp cells^102^. The epithelial compartment of polyp- compared to non-polyp regions of nasal scrapings derived from CRSwNP patients have a reduced cellular diversity characterized by a shift of ciliated and glandular cells to increased basal cell populations. Polyp basal progenitor cells show an aberrant transcriptional profile dominated by extracellular matrix remodeling. Differentiation of basal cells is impaired in the type 2 inflammatory environment of nasal polyps potentially as a result of IL-4/IL-13 and Wnt signaling. When basal cells of polyp- and non-polyp regions are cultured ex vivo, the enhanced response to IL-4/IL-13 is retained^14^ suggesting that epigenetic reprogramming of epithelial stem cells contributes to the chronic type 2 airway inflammation in asthma and CRSwNP, which extends to various locations and cell types (Fig. 2).

### Involvement of type 2 innate lymphoid cells in trained immunity in allergic asthma

Several studies have implicated ILC2 in trained immunity in the context of allergic asthma^47,103,104^ (Fig. 2). ILC2 drive type 2 immune responses at epithelial barriers by producing type 2 cytokines (IL-4, IL-5, IL-13) in response to epithelial alarmins (e.g. IL-33, IL-25)^36,105,106^ or cysLTs^107^, which have been suggested as inducers of trained type 2 immunity (see above). ILC2 derive from common lymphoid progenitor cells and their final differentiation occurs in the fetal liver and peripheral tissues. Therefore, ILC2 residing in the BM are not the source of ILC2 in peripheral tissues, but are rather predicted to have BM-specific functions^108,109^. A recent study has demonstrated that BM-resident ILC2 support hematopoietic recovery following chemotherapy-induced stress and promote hematopoiesis towards the myeloid lineage by secretion of GM-CSF^110^. Whether BM-resident ILC2 can also trigger long-term epigenetic reprogramming of myeloid progenitor cells and thus drive central trained type 2 immunity needs further investigation. Allergen- and IL-33-experienced ILC2 can persist in the lung and draining mediastinal lymph node for several months following allergen exposure. When transferred into naïve mice, IL-33-experienced ILC2 respond more potently to challenge with an unrelated allergen than naïve ILC2 and they display a transcriptional profile similar to memory T cells^47^. While the molecular mechanisms underlying trained ILC2 responses remain largely unknown, a recent study investigating ILC3 enriched at the intestinal mucosal barrier, suggest that metabolic changes as well as an enhanced proliferative capacity contribute to trained immunity in ILC. *Citrobacter rodentium* infection was shown to train ILC3 which persist for months and display a heightened response upon reinfection with the same or an unrelated pathogen. The initial infection induces metabolic rewiring characterized by a shift from glycolysis towards increased tricarboxylic acid (TCA) cycle, fatty acid synthesis and oxidative phosphorylation^111^. Whether a similar metabolic rewiring and increased proliferation might also contribute to trained immunity in ILC2 need further investigation.

Transcriptional and epigenetic reprogramming in ILC2 occurs during repeated exposure to *Alternaria* allergen, resulting in altered gene accessibility of two transcriptional regulators: Bach2, involved in the repression of cytokine genes and the activating transcription factor AP1. Thus, allergen exposure drives the upregulation of two programs, a repressive and a preparedness program in memory-like ILC2. Upon allergen challenge, the preparedness program gets activated triggering AP1 and STAT6 signaling, which induces type 2 inflammation. The balance between both programs (Bach2 and AP1) likely determines and maintains trained immunity in ILC2^112^. In addition, IL-33 induces STAT3 phosphorylation which results in increased ATP synthesis and subsequent generation of S-adenosylmethionine (SAM). As primary substrate of histone methyltransferases, SAM might drive metabolic and epigenetic reprogramming by histone and DNA methylation of innate immune genes in the context of allergic airway inflammation^113–115^.

Finally, trained immunity of ILC2 may help to explain why asthmatic patients are often sensitized to several unrelated allergens. ILC2 can interact with macrophages or myeloid progenitors in the lung or BM, respectively, thus promoting local or central trained type 2 immunity. The nature and mechanisms of the cellular crosstalk that initiate and maintain trained immunity in asthma should be deciphered in future studies.

### Trained immunity in macrophages in allergic and non-allergic asthma

Two recent studies show that macrophages of asthmatic patients maintain an inflammatory transcriptional signature as well as aberrant metabolic and epigenetic profiles during ex vivo differentiation from monocytes.

### Metabolic and epigenetic reprogramming of macrophages in type 2 inflammatory airway disease

The first study suggests that an inflammatory reprogramming of monocyte-derived macrophages (MDM) contributes to the pathogenesis of NSAID-exacerbated respiratory disease (N-ERD), also called aspirin-exacerbated respiratory disease (AERD)^116^, a type 2 inflammatory disease, characterized by CRSwNP, chronic asthma and NSAID intolerance^117^. MDM of N-ERD patients display enhanced inflammatory chemokine (*CXCL1, CXCL8, CCL20*) and eicosanoid (5-HEPE, 5-HETE, LTB_4_) responses associated with reduced methylation of chemokine genes and genes involved in lipid/ acylcarnitine metabolism^53^. Increased expression of enzymes regulating fatty acid oxidation (FAO) (e.g. *Cpt1*, *Hadha)* have also been detected in the lungs of allergen-sensitized mice and pharmacological inhibition of FAO enzymes reduces cell infiltration and cytokine production in the lung during AAI^118^. Whether the metabolic shift towards increased FAO depends on the type 2 inflammatory environment and whether it triggers long-term epigenetic changes in innate immune cells remains to be elucidated. Of note hyporesponsiveness of alveolar macrophages to IL-4 is mediated by impaired glycolysis in the pulmonary environment demonstrating the impact of metabolic changes to macrophage activation and effector functions^119^.

Broadly reduced DNA methylation in N-ERD MDM further suggests that epigenetic reprogramming drives aberrant macrophage responses and chronic type 2 airway inflammation in N-ERD. Of note, DNA methylation has been associated with “true” adaptive immune memory rather than “adaptation” of innate immune cells, which is relatively short in duration and often associated with transient histone modifications^120^. Thus, monocytes and macrophages of asthmatics show a long-lasting epigenetic reprogramming with the potential to drive an functional state that can be referred to as “inflammatory memory”^15,53^. It remains to be established whether inflammatory reprogramming occurs exclusively in monocytes and macrophages derived from myeloid progenitors in the bone marrow of N-ERD patients or whether it can extend to airway resident macrophages. As discussed above, alveolar macrophages can be reprogrammed by viral infection^121^ and potentially by IL-4^45^, suggesting that trained immunity in lung-resident macrophages is induced in the type 2 inflammatory environment in the asthmatic lung. Indeed in inflammatory settings, resident, self-renewing macrophages may be replaced by macrophages derived from infiltrating monocytes with regulatory or proinflammatory roles in asthma^50,122^. Thus, the inflammatory reprogramming of monocytes and their progenitors in the BM is of particular relevance to the induction of chronic airway inflammation (Fig. 2).

### Trained immunity in macrophages during allergic asthma

A second recent study has described an inflammatory imprinting in MDM of house dust mite (HDM)-allergic asthma patients as well as in BMDM of HDM-sensitized mice^16^. In addition to transcriptional reprogramming, MDM from HDM-allergic humans or mice show an overproduction of CCL17 and cysLTs, which play key roles in type 2 inflammation^123,124^. Thus, the inflammatory imprint of macrophages from asthmatic patients appears to be distinct from classical trained immunity in infection or following vaccination. As potential changes in effector functions associated with type 2 immunity have not been systematically addressed in other settings of trained immunity, a potential overlap and functional crosstalk between different trained immunity programs requires further investigation. The upregulation of *Ptgs2* (the gene encoding cyclooxygenase 2) and *Il6*, typically associated with classical trained immunity, in BMDM of HDM-sensitized mice, suggests common features of trained immunity in infection and type 2 inflammation.

Mechanistically, HDM-induced reprogramming of macrophages and their progenitors in the BM depends on TNF production by myeloid cells^16,125^. In addition, metabolic and epigenetic reprogramming may contribute to enhanced chemokine and cysLT responses of HDM-trained macrophages via increased synthesis of 2-hydroxyglutarate (2-HG) and lysine demethylase LSD1 (KDM1)-mediated histone (H3K4/ H3K9) demethylation. 2-HG is known to suppress alpha-ketoglutarate–dependent histone demethylases affecting epigenetic modifications and to promote the stability of the transcription factor hypoxia-inducible factor 1α which is central in the induction of trained immunity^8,126^. In addition, the increased production of adenosine and TCA cycle intermediates by HDM-trained macrophages may contribute to epigenetic and transcriptional reprogramming, thus enhancing “type 2 inflammatory” effector functions. Indeed, adenosine drives inflammatory macrophage functions in the context of AAI and an enrichment of TCA cycle metabolites contributes to trained immunity induced in macrophages stimulated with oxLDL^127,128^.

Despite these insights, the mechanisms of macrophage reprogramming in type 2 inflammation remain only partially defined and further studies are needed to delineate the metabolic and epigenetic pathways that drive the reprogramming of myeloid progenitors in allergic and non-allergic asthma. The upregulation of type 2 associated mediators (particularly cysLTs) wanes one week after the last allergen challenge, while CCL17 and factors characteristic for classical inflammatory macrophage activation (e.g. Il6, Ptgs2) remain upregulated. Thus, inflammatory reprogramming in allergic asthma results in a proinflammatory macrophage phenotype, which may broadly affect subsequent responses to inflammatory or infectious insults. As enhanced chemokine and cytokine production is detected in the lung and BM even after removal of the allergen, it will be important to determine the duration and dynamics of macrophage training in asthma. In addition, the functional relevance of long-term macrophage reprogramming for chronic inflammation and exacerbations in asthma remains to be defined.

### Involvement of trained immunity in mast cells and hematopoiesis during type 2 inflammation

The activation and expansion of mast cells (MCs) in the context of type 2 inflammatory diseases such as asthma are well established^129–131^. For example, MCs proliferate locally in the nasal polyp tissue of N-ERD patients^132^. Classically, MCs are divided into fetal liver-derived constitutive MCs residing in connective tissues and mucosal MCs arising from BM progenitor cells^133,134^. Upon activation, MCs rapidly release granules containing histamine and synthesize lipid mediators (e.g. cysLTs and prostaglandin D_2_), thus driving immediate responses such as vascular leakage, bronchoconstriction and itch, and promoting the induction of type 2 inflammation^135,136^. Additionally, MCs have been shown to secrete IL-4 and IL-13, however, the functional roles of mast cell-derived type 2 cytokines in trained immunity during allergy or helminth infection warrants further investigation^137,138^. During experimental AAI triggered by ovalbumin sensitization (i.p.), mast cell progenitors are recruited to the lung via vascular cell adhesion molecule 1 (VCAM-1), which contributes to airway remodeling and inflammation^139,140^. LTB_4_, a major lipid mediator produced by alveolar macrophages and neutrophils, is highly chemotactic for MC progenitors and the responsiveness to LTB_4_ inversely correlates with MC maturation^141^. MCs are long-lived cells, however, so far studies investigating trained immunity in mast cells have focused predominately on endotoxin priming and infection models^142^. Potential trained immunity programs in constitutive MCs and/or BM-derived mucosal MCs in the context of type 2 inflammation remains to be elucidated. The finding that DNA methylation affects MC responses in acute and chronic dermatitis models may imply that epigenetic reprogramming of MCs contributes to trained immunity in allergic diseases^143^. Since trained immunity has been described for a variety of immune cells involved in type 2 inflammation, it seems likely that MCs as well as their progenitor cells in the BM can undergo epigenetic reprogramming upon allergen exposure and contribute to the enhanced reaction to subsequent allergen challenge. Several studies indicate that hematopoiesis, especially granulopoiesis is altered in asthmatic patients and in mouse models of type 2 immunity^144–148^. TSLP, IL-25 and IL-33 affect HSPC activation and differentiation^149–152^ and TSLP can promote basophil hematopoiesis, which results in transcriptionally and functionally altered basophils with an enhanced capacity to drive type 2 inflammation^149^. CD34^+^ progenitor cells are increased at sites of allergic inflammation^153,154^ and cells co-expressing CD34^+^/IL-5^+^ and CD34^+^/IL-13^+^ are present in the sputum of asthmatic patients, suggesting that the expansion of type 2 primed progenitor cells may contribute to allergic inflammation^155^. Whether allergens, alarmins or type 2 cytokines can induce central trained immunity by affecting hematopoiesis and by triggering long-term changes in HSPCs in the context of type 2 inflammation needs further investigation.

### Crosstalk between innate and adaptive immune cells in trained type 2 immunity

The innate and adaptive immune system are closely linked to each other, particularly by dendritic cells (DCs) which prime naïve T cells and initiate the activation and formation of memory T cells^156,157^. It was shown that memory-like DCs are induced in response to fungal infection^158^, however the contribution of trained DCs to type 2 immunity are only beginning to emerge (see below)^104,159^. Several studies suggest that a crosstalk between both arms of the immune system coordinates the initiation and maintenance of immune memory and trained immunity. For example, Th2 cells may be responsible for the increased type 2 cytokine levels found in the BM of HDM-challenged mice^16^ and thus drive the inflammatory reprogramming of myeloid progenitor cells during AAI. Indeed, the induction of long-lasting changes in alveolar macrophages during respiratory viral infection is T cell dependent demonstrating a contribution of adaptive immune cells to the induction of trained immunity^121^. Additionally, memory-like ILC2 secrete elevated levels of IL-5 and IL-13 and promote the differentiation of naïve CD4^+^ T cells into Th2 cells, thus providing a possible link between trained immunity and adaptive immune memory^47^. Once an allergen-specific T- and B cell memory has been established, trained macrophages, DCs, ILC2 and epithelial cells may maintain the type 2 inflammatory environment that promotes the recruitment and activation of memory Th2 cells. However, the cellular crosstalk and series of events that govern and link trained immunity and adaptive immune memory require further investigation.

### Therapeutic targeting of the inflammatory memory in type 2 inflammation

As discussed above, inflammatory memory of innate immune cells and epithelial cells may initiate and promote type 2 inflammation and aggravate disease progression. Thus, targeting the different mechanisms involved in the inflammatory reprogramming of innate immune cells and epithelial cells represents a promising strategy to improve currently available therapies. Elucidating the effects of approved treatments against type 2 inflammatory diseases on innate immune training may deepen our understanding about trained type 2 immunity in humans and guide treatment rationales for particular disease endotypes. IL-4 and IL-13 signaling results in the activation of STAT6, a key transcription factor involved in type 2 immune responses^160,161^. Treatment with a peptide inhibiting STAT6, STAT6-IP, reduces airway hyperresponsiveness und lung inflammation^159^ suggesting the therapeutic application of this peptide. The anti-inflammatory effects of STAT6-IP are at least partially explained by reprogramming of DCs into a tolerogenic phenotype, expressing IDO and TGFβ, which promotes the induction of regulatory T cells. The tolerogenic phenotype of DCs is maintained when allergen-sensitized DCs isolated from STAT6-IP vaccinated mice are transferred into naïve mice suggesting that STAT6-IP treatment can induce a stable anti-inflammatory imprint in myeloid cells^162^ that contributes to its therapeutic effects on type 2 inflammation.

Although requiring long-term administration for years and despite significant numbers of non-responders, allergen-specific immunotherapy (AIT) currently represents a particularly successful treatment against allergic diseases. A recent study suggests that the induction of a tolerance program in innate immune cells contributes to the therapeutic effects of AIT^104^. Thus, AIT alters the composition of monocytes, ILC and DCs and numbers of ILC2 and ILC3 remain reduced for extended periods of time following AIT. In addition to inducing a shift towards anti-inflammatory monocytes, AIT results in an increase in plasmacytoid DCs (pDCs) and a decrease in CD1c^+^ myeloid DCs (mDCs)^104^. As pDCs are implicated in antiviral immunity while mDCs drive type 2 immune responses^163,164^, AIT may alter the composition of the myeloid compartment to limit type 2 inflammation and restore a homeostatic innate immune status.

Leukotrienes are key drivers of pathological type 2 inflammation^37,123^ and pharmacological agents targeting cysLT receptors have been developed as therapeutics against asthma. Montelukast is a cysLT receptor antagonist, which reduces eosinophilia and airway hyperresponsiveness in asthmatic patients^165–167^. As MDM of asthmatic patients as well as BMDM of HDM-sensitized mice show enhanced cysLT synthesis^16^, potential therapeutic effects of montelukast on the functionality of type 2 trained macrophages should be investigated.

Dupilumab, a monoclonal antibody targeting IL-4Rα is successfully used as a treatment against type 2 inflammatory diseases, including atopic dermatitis, allergic asthma and N-ERD^168^. The efficacy of Dupilumab correlates with enhanced baseline eosinophil levels in asthmatic patients as well as with reduced cysLT and increased prostaglandin E_2_ levels implying that Dupilumab efficiently downregulates the activation and recruitment of myeloid cells involved in type 2 inflammation^169,170^. IL-4Rα is also expressed on airway epithelial cells and its expression levels are increased in the epithelia of asthmatic patients^14,171,172^. Thus, it would be relevant to study therapeutic effects of Dupilumab on the reprogramming of epithelial stem cells and HSPCs in type 2 inflammation. In conclusion, there are several promising strategies to target the inflammatory memory in chronic type 2 inflammation and future studies might decipher further candidates targeting key mechanisms of trained type 2 immunity in asthma or CRSwNP.

## Conclusions and remaining questions

The phenomena of “trained immunity” and “inflammatory memory” may help to explain multiple characteristics of host defense and inflammation in type 2 immune responses. For example, non-allergic asthma and nasal polyposis are driven by an intricate interplay of innate immune cell subsets, including ILC, macrophages, eosinophils and neutrophils^53,173–175^, which may have undergone long-term reprogramming in response to pathological triggers such as viral or bacterial infection or exposure to pollutants or molds^93,176–178^. In addition, an epigenetic and transcriptional reprogramming of airway epithelial cells^14^ may contribute to chronic type 2 airway inflammation, by providing continuous inflammatory cues in the form of lipid mediators or alarmins, which recruit, expand and activate innate immune cells. In the context of asthma and nasal polyposis, it will be important to distinguish whether chronic inflammation is driven by a long-term reprogramming in one or several cell types or by the continuous exposure to allergens or other inflammatory insults. Enhanced inflammatory responses as well as transcriptional and epigenetic changes in ex vivo cultured cells from patients with asthma or nasal polyps imply the involvement of trained immunity^14,16,53^. However, the duration of the inflammatory memory observed in innate immune cells of patients suffering from chronic type 2 inflammation can hardly be inferred from such experiments. Mouse models of allergic and non-allergic asthma may aid to assess the duration and stability of inflammatory memory responses triggered by allergens or potential infectious triggers such as molds or respiratory viruses. Such models also allow for functional and mechanistic studies of trained type 2 immunity in the absence of functional T- and B cells. This will hopefully clarify a potential contribution of the adaptive arm of type 2 immunity to the maintenance and propagation of trained immunity. In turn, the induction of trained immunity might influence adaptive immune memory in chronic type 2 inflammation. Recent studies investigating inflammatory memory responses in airway epithelial cells, ILC2 and monocytes/macrophages should be complemented by studies characterizing the crosstalk between these cell types as well as with other key cells involved in type 2 inflammation, including Th2 cells, mast cells and eosinophils.

Current single cell sequencing technologies with the potential to assess chromatin accessibility and transcriptional profiles in innate immune cells and their progenitors will be highly instrumental in defining the mechanisms of trained immunity in settings of asthma or helminth infection. In particular, single cell RNAseq and ATACseq analyses will enable the detailed characterization of transcriptional and epigenetic signatures in innate immune cell- and HSPC subsets that expand and persist in the tissue or BM following the initial induction of a type 2 immune response. In combination with mouse strains lacking suspected mechanisms of trained type 2 immunity in the myeloid or hematopoietic compartment, these techniques should greatly facilitate the mechanistic and functional elucidation of trained immunity and inflammatory memory responses in helminth infection or type 2 inflammatory diseases such as allergy and asthma.

Current evidence suggests that trained immunity during helminth infection or asthma can be triggered directly, i.e. by modulation of innate immune cell function by helminth products^30^ or allergens^16^, or indirectly by alarmins and cytokines produced by damaged epithelial cells^14^ or activated innate immune cells^16,31^. The exact molecular characteristics of allergenic or helminth-derived triggers of trained immunity as well as their cellular targets remain to be further defined.

Finally, given that the susceptibility to chronic type 2 inflammatory diseases is increased in children of affected parents^179^, it will be relevant to study a potential vertical transmission of trained immunity^18,19^ in allergy and asthma. A detailed understanding of the mechanisms, functions and potential inheritance of trained immunity in helminth infection and chronic type 2 inflammation should foster the development of preventive and therapeutic strategies targeting these common global health burdens.
