# Correction to: Trained immunity in type 2 immune responses

**DOI:** 10.1038/s41385-022-00568-x

**Published:** 2022-09-16

**Authors:** Franziska Hartung, Julia Esser-von Bieren

**Affiliations:** Center of Allergy and Environment (ZAUM), Technical University of Munich and Helmholtz Center Munich, 80802 Munich, Germany

Correction to: *Mucosal Immunology* 10.1038/s41385-022-00557-0, published online 05 September 2022

The original version of this article unfortunately contained a mistake in the figure legends as the following statement was missing “Created with BioRender.com”. The original article has been corrected.

